# The Association of Broadband Internet Access and Telemedicine Utilization in rural Western Tennessee: an observational study

**DOI:** 10.1186/s12913-021-06746-0

**Published:** 2021-08-03

**Authors:** Jacob K. Quinton, Michael K. Ong, Sitaram Vangala, Anna Tetleton-Burns, Ashley Webb, Catherine Sarkisian, Alejandra Casillas, Preeti Kakani, Maria Han, Claude J. Pirtle

**Affiliations:** 1 Division of General Internal Medicine and Health Services Research, Department of Medicine, University of California Los Angeles, Los Angeles, CA USA; 2 Department of Health Policy & Management, Fielding School of Public Health, University of California Los Angeles, Los Angeles, CA USA; 3 VA Center for the Study of Healthcare Innovation, Implementation and Policy (CSHIIP), Los Angeles, CA USA; 4 Department of Virtual Care, West Tennessee Healthcare, Jackson, TN USA; 5 Geriatrics Research Education and Clinical Center, Veterans Affairs Greater Los Angeles Healthcare System, Los Angeles, CA USA; 6 David Geffen School of Medicine, University of California Los Angeles, Los Angeles, CA USA

## Abstract

**Background:**

Broadband access has been highlighted as a national policy priority to improve access to care in rural communities.

**Objective:**

To determine whether broadband internet availability was associated with telemedicine adoption among a rural patient population in western Tennessee.

**Methods:**

Observational study using electronic medical record data from March 13th, 2019 to March 13th, 2021. Multivariable logistic regression incorporating individual-level characteristics with broadband availability, income, educational attainment, and primary care physician supply at the zip code level, and rural status as determined at the county level.

**Setting:**

Single health system in western Tennessee.

**Participants:**

Adult patients with one or more in-person or remote encounter in a health system in western Tennessee and residing in western Tennessee between March 13th, 2019 and March 13th, 2021 (*N* = 54,688).

**Outcome measures:**

Completion of one or more video encounters in the year following March 13th, 2020 (*N* = 3199; 7%). Our primary characteristic of interest was the proportion of residents in each zip code with access to the internet meeting the Federal Communications Commission definition of broadband access, adjusting for age, gender, race, income, educational attainment, insurance type, rural status, and primary care provider supply.

**Results:**

Patients in a rural western Tennessee health system were predominantly white (79%), residing in rural zip codes (73%) with median household incomes ($52,085) less than state and national averages. Patients residing in a zip code where there is 80 to 100% broadband access compared to 0 to 20% were more likely in the year following March 13th, 2020 to have completed both telemedicine and in-person visits ([OR; 95% CI] 1.57; 1.29, 1.94), completed only telemedicine visits (2.26; 1.71, 2.97), less likely to have only completed in-person visits (0.81; 0.74, 0.89), but no more or less likely to have accessed no care (1.07; 0.97, 1.18).

**Discussion:**

The availability of broadband internet was shown to be one of many factors associated with the utilization of telemedicine for a rural, working-class community after March 13th, 2020.

**Conclusions:**

Access to broadband internet is a determinant of access to telemedicine for patients in rural communities and should be a priority for policymakers interested in improving health and access to care for rural patients.

**Supplementary Information:**

The online version contains supplementary material available at 10.1186/s12913-021-06746-0.

## Introduction

Rural areas account for 97% of the landmass of the United States (US) and only 20% of the population, and rural health care is characterized by the sequelae of the low population density which defines rural life [1]. Rural areas lag compared to urban areas in access to technology, including broadband-speed internet [2]. Rural Americans have less access to primary and specialty care as well as decreasing access to tertiary care facilities as critical access hospitals have continued to close, leaving rural communities older, sicker, and more isolated than urban communities [3, 4].

The incorporation of telemedicine services, narrowly defined as remote synchronous audio-video encounters, has been considered a possible resolution to access to care issues for rural patients [2, 5–7]. Despite early demonstrations of telemedicine effectiveness including ‘hub-and-spoke’ models of access to specialist care [8], uptake of telemedicine services had been relatively limited across the general population before the COVID-19 pandemic. The rapid incorporation of telemedicine into ambulatory care beginning in March 2020 has been widespread, with a rapid transition to telemedicine now attenuating to a ‘blend’ of telemedicine and in-person care [7]. The western region of Tennessee, or westernmost grand division, is an agricultural region geographically and culturally distinct from the other grand divisions of Tennessee (middle and east Tennessee), anchored by the city of Memphis in the southwest and the Mississippi and Tennessee rivers forming the western and eastern borders, respectively [9]. The 18 counties of western Tennessee have a median household income of $46,769 with 17% of the population living in poverty [10]. The primary provider of health care in rural western Tennessee is West Tennessee Healthcare (WTH) accounting for about 163,000 outpatient yearly visits in 2019 [11].

The primary objective of our observational study was to determine the association between utilization of telemedicine amongst a predominantly working-class rural population in western Tennessee with zip code-level broadband access estimates in the year following March 13th 2020, while incorporating individual-level demographic and clinical information and income, educational attainment, and primary care physician availability at the zip-code level, and rural status at the county level.

## Methods

We abstracted electronic health record data from all patients greater than 18 years of age (*N* = 61,521) who presented for one or more ambulatory encounters at WTH in the years before and after restrictions on large gatherings and cessation of non-essential travel were announced by the state of Tennessee on March 13th, 2020 [12]. We excluded all patients residing outside of the geographic region of western Tennessee (6047) as well as those living in Shelby County (180). Patients residing in Shelby County typically reside in the city of Memphis and are non-representative of rural access to care issues. Conversations with WTH operations staff revealed a telemedicine champion cardiologist who enthusiastically transitioned encounters to telemedicine (50% of visits in the following year), and so patients seen by this specialty were excluded (735) for a final analytic sample (*N* = 54,559). Median household income data and average educational attainment (defined by the proportion of individuals in each zip code with a bachelor’s degree) were obtained from the US Census [10], rural status and primary care health professional shortage area (pcHPSA) score were obtained from the Health Resources and Services Administration (HRSA) [13], and the proportion of each county with access to internet meeting the Federal Communications Commission definition of broadband (download speeds of 25 megabytes per second [mbps], and upload speeds of 3 mbps) [14] from a publicly available website (Table 1) [15].

**Table 1:** Characteristics of patients in western Tennessee using both telemedicine and in-person care, only telemedicine, only in-person, or no office-based care after March 13th, 2020

	All patients with at least one in-person visit before March 13th, 2020	All patients using in-person care and telemedicine after March 13th, 2020	All patients using only telemedicine after March 13th, 2020	All patients using only in-person care after March 13th, 2020	Patients getting no office-based care after March 13th, 2020
	N (%)	N (%)	N (%)	N (%)	N (%)
**Total Population**	40,711 (75)	3199 (5)	993 (2)	32,430 (60)	18,930 (35)
**Mean Age (SD)**	56 (19)	54 (19)	51 (19)^a^	55 (19)^a^	53 (19)^a^
**Race**					
White	32,319 (79)	1481 (86)^a^	434 (88)^a^	15,449 (79)^a^	14,955 (79)^a^
Black	7387 (18)	229 (13)^a^	49 (10)^a^	3744 (19)^a^	3365 (18)^a^
Other	454 (1)	10 (1)^a^	3 (1)^a^	195 (1)^a^	246 (1)^a^
Missing	541 (1)	9 (1)^a^	5 (1)^a^	173 (1)^a^	354 (2)^a^
**Gender**					
Female	24,270 (60)	1091 (63)	283 (58)	11,526 (59)^a^	11,525 (59)
**Patient Language**					
English	40,541 (100)	1723 (99)^a^	488 (99)	19,513 (99)^a^	18,817 (99)^a^
Other	36 (< 1)	2 (< 1)^a^	0	17 (< 1)^a^	17 (< 1)^a^
Unknown	125 (< 1)	4 (1)^a^	3 (< 1)	31 (< 1)^a^	87 (1)^a^
**Insurance Type**					
Commercial	15,770 (39)	755 (44)^a^	259 (53)^a^	7189 (37)^a^	7567 (40)^a^
Medicaid	3404 (8)	103 (6)^a^	28 (6)^a^	1566 (8)^a^	1707 (9)^a^
Medicare	10,959 (27)	506 (29)^a^	117 (20)^a^	5860 (30)^a^	4476 (24)^a^
Medicare Adv.	7022 (17)	260 (15)^a^	50 (10)^a^	3777 (19)^a^	2935 (16)^a^
Other	341 (1)	17 (1)^a^	3 (1)^a^	165 (1)^a^	156 (1)^a^
Self-Pay	3215 (8)	88 (5)^a^	34 (7)^a^	1005 (5)^a^	2089 (11)^a^
**Broadband Access**					
80 to 100%	29,535 (72)	1656 (69)^a^	655 (66)^a^	23,542 (73)^a^	14,016 (73)^a^
60 to 80%	5965 (15)	335 (14)^a^	163 (16)^a^	4916 (15)^a^	2611 (14)^a^
40 to 60%	3203 (8)	223 (9)^a^	101 (10)^a^	2429 (7)^a^	1563 (8)^a^
20 to 40%	510 (1)	20 (1)^a^	10 (1)^a^	431 (1)^a^	262 (1)^a^
0 to 20%	1601 (4)	149 (6)^a^	66 (7)^a^	1164 (4)^a^	822 (4)^a^
**HRSA Category**					
Non-Rural	516 (1)	9 (1)^a^	3 (1)^a^	279 (1)^a^	225 (1)^a^
Partially Rural	10,340 (25)	742 (43)^a^	218 (44)^a^	4361 (23)^a^	5019 (27)^a^
Rural	29,855 (73	978 (56)^a^	270 (55)^a^	14,921 (76)^a^	13,686 (72)^a^
**Mean pcHPSA (SD)**	15 (3)	16 (3)^a^	16 (3)^a^	15 (3)^a^	15 (3)
**Mean Median HH Income (SD)**	52,085 (20,797)^a^	46,838 (18,146)^a^	45,565 (17,036)^a^	53,190 (21,263)^a^	51,549 (20,485)^a^
**Mean proportion with BA (SD)**	23 (13)	21 (11)^a^	20 (10)^a^	23 (13)^a^	23 (12)^a^

^a^*p* < 0.05, the reference group consists of all patients seen at least once between March 13th, 2019 and March 13 ^th^, 2021

We constructed a multivariable regression model including age, gender, race, primary insurance, rural status, primary care provider supply, and a multi-level variable of quintile of broadband access with the lowest proportion of access as the reference group (i.e. 0–20%). For the remaining zip-code level variables we also constructed quintiles using the lowest quintile (0–20%) as the reference group. We hypothesized patients residing in zip codes with greater broadband access would be more likely to use telemedicine than those from zip codes with reduced access. Our primary outcome was the completion of both telemedicine and in-person visits in the year since March 13th, 2020. We conducted sensitivity analyses with the same covariates with patients who had completed only telemedicine visits, only in-person visits, and those completing no visits since March 13th, 2020. We additionally conducted a sensitivity analysis analyzing only the patient cohort seen by the cardiology telemedicine champion (Additional file 1). For unadjusted comparisons we used two-way t-tests for continuous variables and chi-squared tests for categorical variables. All significance testing was at the level of *p*-values < 0.05, and all analyses were performed using STATA 16c.

## Results

In the year following March 13th, 2020, of patients completing in-person encounters in the year prior 3199 patients completed both telemedicine and in-person visits, 993 patients completed only telemedicine visits, 32,430 patients completed only in-person visits, and 18,930 patients completed no visits (Table 1). Patients with one or more visit in the year prior to the safer at home order differed from those using both in-person care and telemedicine, only telemedicine or in-person care, and receipt of no care in the year after by the proportion of residents with broadband access, race/ethnicity, primary insurance type, rural status, primary care provider supply, median income, or educational attainment in unadjusted comparisons (Table 1).

**Fig. 1:**
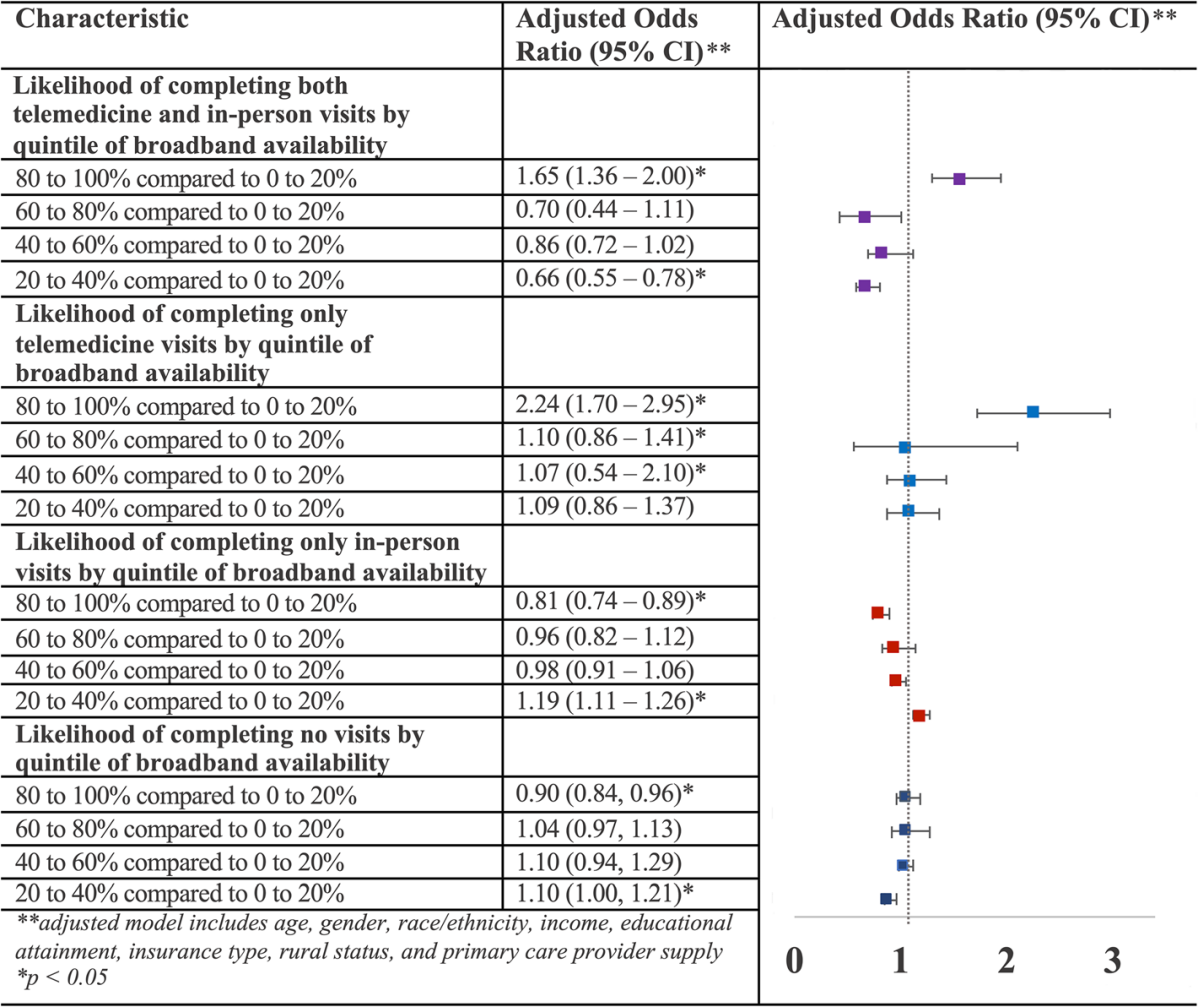
Broadband internet access association with completion of remote and in-person visits or receiving no care in the year after March 13th, 2020

Patients utilizing both modalities as well as those utilizing only telemedicine visits in the year after March 13th, 2020 compared to all patients in the study period were more likely to reside in areas with 80–100% access to broadband internet than those with 0–20% access and were less likely to access in-person care only in adjusted comparisons controlling for age, gender, primary insurance type, race/ethnicity, income, educational attainment, and primary care provider supply (Fig. 1).

Patients utilizing both telemedicine and in-person visits (as well as only telemedicine visits) were more likely to be White than Black, and to have commercial insurance compared to Medicaid, traditional Medicare or Medicare Advantage in adjusted comparisons (Table 2). They were also more likely to reside in partially rural areas than rural areas, and areas of increased primary care provider shortage. Patients utilizing both modalities or only telemedicine were more likely to reside in areas in a lower quintile of median household income, whereas those living in areas with a higher quintile of median household income were more likely to utilize in-person care alone.

**Table 2:** Multivariable regression estimates of characteristics of patients in western Tennessee by remote and in-person care accessed after March 13th, 2020

Characteristic	Adjusted Odds Ratio (95% CI)			
	Both telemedicine and in-person care	Only telemedicine	Only in-person	No ambulatory care received
**Age**				
Age, by year	1.00 (1.00, 1.00)^a^	0.99 (0.99, 1.00)^a^	1.00 (1.00, 1.00)^a^	1.00 (1.00, 1.00)^a^
**Race**				
Black versus White	0.83 (0.72, 0.97)^a^	0.54 (0.42, 0.70)^a^	1.13 (1.07, 1.19)^a^	0.93 (0.88, 0.98)^a^
Other versus White	0.44 (0.24, 0.84)^a^	0.86 (0.44, 1.67)	1.10 (0.93, 1.30)	1.00 (0.84, 1.19)
Missing versus White	0.68 (0.42, 1.09)	1.37 (0.84, 2.25)	0.92 (0.81, 1.06)	1.12 (0.98, 1.29)
**Gender**				
Female	0.83 (0.75, 0.92)^a^	0.93 (0.80, 1.08)	1.09 (1.05, 1.14)^a^	0.95 (0.91, 0.98)^a^
**Insurance**				
Medicaid versus commercial	0.65 (0.53, 0.80)^a^	0.44 (0.31, 0.61)^a^	1.22 (1.13, 1.31)^a^	0.92 (0.85, 0.99)^a^
Medicare versus commercial	0.83 (0.71, 0.96)^a^	0.70 (0.55, 0.89)^a^	1.01 (0.95, 1.07)	1.04 (0.98, 1.11)
Medicare advantage versus commercial	0.64 (0.54, 0.75)^a^	0.50 (0.38, 0.65)^a^	1.18 (1.11, 1.25)^a^	0.94 (0.88, 1.00)^a^
Self-Pay versus commercial	0.59 (0.47, 0.75)^a^	0.73 (0.55, 0.96)^a^	0.63 (0.58, 0.67)^a^	1.77 (1.64, 1.91)^a^
Other versus commercial	1.29 (0.80, 2.07)	1.06 (0.52, 2.16)	0.95 (0.77, 1.18)	1.02 (0.82, 1.27)
**Rural Status**				
Partially versus rural county	2.58 (2.31, 2.89)^a^	2.64 (2.25, 3.11)^a^	0.61 (0.58, 0.64)^a^	1.29 (1.22, 1.35)^a^
Non-rural versus rural county	0.75 (0.43, 1.30)	0.69 (0.26, 1.88)	1.09 (0.93, 1.28)	0.95 (0.80, 1.11)
**pcHPSA score**	1.06 (1.03, 1.08)^a^	0.96 (0.93, 0.99)^a^	0.98 (0.97, 0.99)^a^	1.01 (1.00, 1.02)
**Quintile of median household income**	0.86 (0.80, 0.92)^a^	0.85 (0.78, 0.93)^a^	1.09 (1.06, 1.12)^a^	0.95 (0.93, 0.98)^a^
**Quintile by percent of population with bachelor’s degree**	1.04 (1.01, 1.06)^a^	0.92 (0.85, 1.00)	0.96 (0.94, 0.99)^a^	1.04 (1.01, 1.06)^a^

^a^*p* < 0.05, the reference group consists of all patients seen at least once between March 13, 2019 and March 13, 2021

## Discussion

Telemedicine has been proposed by policymakers and clinicians in rural areas as a resolution to access to care barriers in rural communities, and both groups highlight the importance of access to broadband internet as a prerequisite to telemedicine use. In the first year of the COVID-19 pandemic, there was an unexpected opportunity to test this hypothesis, and we found residents residing in areas with increased broadband access to be more likely to utilize telemedicine than those with limited access to broadband. We did not find, however, that those living in areas of higher versus lower broadband availability were more or less likely to not utilize care, indicating that telemedicine may support previously planned care but may not induce additional demand for services.

We found disparities amongst those accessing telemedicine by insurance benefit design, and self-reported race, persisting after adjustment for income, primary care provider access, and educational attainment. We purposely analyzed Medicare Advantage (MA) patients separately from traditional Medicare as authors had noted media attention regarding MA plans promoting the flexibility of their benefit design in rapidly transitioning care modalities. This flexibility may promote telemedicine uptake in other populations but was not found in our study. We also found that there was an inverted relationship between income and likelihood of engaging in telemedicine care compared to the hypothesized relationship. From prior literature we had hypothesized that there would be a direct relationship between income and likelihood of utilization of telemedicine. We found an inverse relationship, and suggest that this finding may be due to local unobserved factors in this single health system and must be taken into consideration of the broader literature and confirmed in further studies. Our finding regarding increased educational attainment being associated with increased likelihood of a telemedicine encounter was congruent with prior literature and our priors. Finally, while the majority of the patients were White, patients who were Black reported a lower likelihood of using telemedicine and in-person care or telemedicine alone and a higher likelihood of only using in-person care. Whether this represents preference or structural barriers to care associated with systemic discrimination is beyond the scope of our study, but the differences may be explored in future studies.

Our study has several limitations. First, there were few patients in our sample (1%) residing in zip codes with 20–40% of broadband access and we found a countervailing association amongst this group compared to other quintiles. Second, while the sample is principally (more than 75%) rural there were a relatively small (7%) proportion of patients who utilized telemedicine, which may be due to quickly attenuated restrictions in response to COVID-19 in Tennessee compared to other states. Third, we did exclude results from a telemedicine champion whose patients had adopted telemedicine visits at 10 times the average of other clinicians, although we did analyze these results separately as a sensitivity analysis.

In conclusion, access to broadband internet was associated with utilization of telemedicine in a rural population in western Tennessee after adjusting for income, educational attainment, primary care clinician supply, rural status, and primary insurance type. This supports the assertion that access to broadband internet is an important determinant of access to care in rural populations. Also important for policymakers, we found racial disparities in utilization of telemedicine, which will be important to consider how best to ameliorate. Finally, we found primary insurance type to be associated with telemedicine utilization. Policymakers in Tennessee and nationally should consider how best to incentivize telemedicine among publicly insured groups and among minority populations.

## Supplementary Information

**Additional file 1: Appendix Table 1.** Characteristics of characteristics of patients in western Tennessee among all patients, as well as those seen by cardiologist telemedicine champion after March 13th, 2020.

## Data Availability

The datasets generated and/or analyzed during the current study are not publicly available due to containing patient identifying information from electronic health record data, but are available from the corresponding author on reasonable request.
