# Supplementary material for: The Association of Broadband Internet Access and Telemedicine Utilization in rural Western Tennessee: an observational study
Source: BMC Health Serv Res. 2021 Aug 3;21:765. doi: 10.1186/s12913-021-06746-0 (PMC8329625; doi:10.1186/s12913-021-06746-0)
Supplement: Supplementary file 1 — Additional file 1: Appendix Table 1. Characteristics of characteristics of patients in western Tennessee among all patients, as well as those seen by cardiologist telemedicine champion after March 13th, 2020. [file 12913_2021_6746_MOESM1_ESM.docx]

**Appendix Table 1:** Characteristics of characteristics of patients in western Tennessee among all patients, as well as those seen by cardiologist telemedicine champion after March 13^th^, 2020

|  | Main Analysis | Telemedicine Champion |
| --- | --- | --- |
|  | **N (%)** | **N (%)** |
| **Total Population** | 55,387 (100) | 699 (1) |
|  |  |  |
| **Mean Age (SD)** | 54 (19) | 54 (19) |
| **Race** |  |  |
| White | 32,319 (79) | 1,481 (86)* |
| Black | 7,387 (18) | 229 (13)* |
| Other | 454 (1) | 10 (1)* |
| Missing | 541 (1) | 9 (1)* |
| **Gender** |  |  |
| Female | 33,255 (60) | 311 (58) |
| **Patient Language** |  |  |
| English | 55,080 (100) | 531 (99)* |
| Other | 58 (< 1) | 1 (< 1)* |
| Unknown | 215 (< 1) | 0 (1)* |
| **Insurance Type** |  |  |
| Commercial | 22,171 (40) | 169 (32)* |
| Medicaid | 5,262 (10) | 63 (12)* |
| Medicare | 13,515 (24) | 152 (29)* |
| Medicare Adv. | 9,476 (17) | 123 (23)* |
| Other | 461 (1) | 2 (< 1)* |
| Self-Pay | 4,502 (8) | 23 (4)* |
| **Broadband Access** |  |  |
| 80 to 100% | 29,535 (72) | 1,656 (69)* |
| 60 to 80% | 5,965 (15) | 335 (14)* |
| 40 to 60% | 3,203 (8) | 223 (9)* |
| 20 to 40% | 510 (1) | 20 (1)* |
| 0 to 20% | 1,601 (4) | 149 (6)* |
| **HRSA Category** |  |  |
| Non-Rural | 771 (1) | 2 (1)* |
| Partially Rural | 14,135 (26) | 358 (67)* |
| Rural | 40,481 (73) | 172 (32)* |
| **Mean pcHPSA (SD)** | 15 (3) | 15 (3) |
| **Mean Median HH Income (SD)** | 51,977  (20,642) | 45,777  (17,171)* |
| **Mean proportion with BA (SD)** | 23 (12) | 18 (7)* |
